# GLP-1 receptor agonists for smoking cessation: a narrative review of weight management potential

**DOI:** 10.1097/MS9.0000000000004725

**Published:** 2026-01-27

**Authors:** Ghazi Uddin Ahmed, Eiman Zehra, Sana Rasheed, Syed Owais Akhtar, Ahmed Asad Raza, Khunsha Mujaddadi, Abedin Samadi

**Affiliations:** a Department of Medicine, Jinnah Sindh Medical University, Karachi, Pakistan; b Department of Medicine, Kabul University of Medical Sciences, Abu Ali Sina, Kabul, Afghanistan

**Keywords:** GLP-1 receptor agonists, metabolic health, nicotine dependence, smoking cessation, weight gain

## Abstract

Smoking cessation is a major public health goal, yet concerns about post-cessation weight gain remain a significant barrier for many smokers. The metabolic and behavioral changes following nicotine withdrawal frequently lead to increased caloric intake and reduced energy expenditure, potentially triggering relapse. Glucagon-like peptide-1 receptor agonists (GLP-1 RAs), originally developed for type 2 diabetes and later approved for weight management, have recently been investigated as a potential strategy to address both nicotine dependence and weight control. Emerging evidence from preclinical studies and limited clinical trials suggests that GLP-1 RAs may reduce nicotine cravings and intake by modulating central reward pathways while simultaneously curbing appetite and supporting weight loss. This dual mechanism may improve quit success rates and mitigate the psychological burden of post-cessation weight gain. However, the current human evidence base is limited, with small sample sizes, short follow-up periods, and reliance on preliminary data. While the pharmacological profile and cardiometabolic benefits of GLP-1 RAs make them a promising candidate for adjunctive use in smoking cessation, more rigorous randomized controlled trials are required to confirm efficacy, evaluate safety, and establish clinical guidelines. This narrative review synthesizes existing evidence to highlight both the potential and the current uncertainties surrounding GLP-1 RAs as dual-target pharmacotherapy for smoking cessation and weight management.

## Introduction

Quitting smoking yields immense health benefits, yet many smokers hesitate because of anticipated weight gain^[1]^. This association has been well established. Thus, for many people, concerns about weight changes can overshadow efforts to quit and jeopardize chances of long-term success. A meta-analysis demonstrated that smoking cessation is generally followed by a weight increase of 4–5 kilograms over the course of a year, with the majority occurring in the initial 3 months^[2]^. Additionally, another study reported that most quitters gained around 2–7 kilograms within the first few months; however, some gained more than ~ 9 kilograms^[3]^.

Cigarette smoking is associated with numerous chronic diseases and plays a significant role in cardiovascular disease, accounting for approximately one-quarter of related deaths. Furthermore, smoking is implicated in one-third of cancer-related deaths, including nearly 90% of lung cancers. Smoking is also a major contributor to chronic obstructive pulmonary disease (COPD) and is responsible for up to 80% of associated deaths^[4–7]^.

**HIGHLIGHTS**

- Glucagon-like peptide-1 receptor agonists (GLP-1 RAs) may aid smoking cessation by reducing nicotine cravings.
- These agents also prevent weight gain common after quitting smoking.
- They act on reward and appetite pathways in the central nervous system.
- GLP-1 RAs support dual goals: abstinence and metabolic health.
- More clinical trials are needed to confirm their efficacy in cessation.

Consequently, this levies a heavy toll on both the economy and the health care system. In the U.S., the burden of smoking was estimated to surpass $600 billion in 2018, with lost productivity contributing approximately $370 billion to this total expenditure^[8]^. These harms make smoking cessation crucial for improving public health.

Many smokers face the classic trade-off between the fear of weight gain and the well-established health risks of smoking. Quitting is notoriously difficult because nicotine addiction is multifactorial. In fact, even after abstaining for a year, many smokers still relapse, with the annual relapse rate estimated at around 10%^[9]^. Concerns about gaining weight are cited as a significant deterrent to quit attempts and may increase the risk of relapse^[10]^. Current treatment options, such as nicotine replacement therapy (NRT), varenicline, and bupropion, help reduce the risk of relapse. Although they may postpone weight gain during smoking cessation, their long-term effectiveness is limited^[11,12]^.

Therefore, the development of novel intervention strategies for smoking cessation is crucial to effectively reduce relapse and address weight-related challenges. Recently, glucagon-like peptide-1 receptor agonists (GLP-1 RAs) have emerged as promising agents. GLP-1 RAs (e.g., exenatide, liraglutide, semaglutide) are incretin mimetics originally developed for type 2 diabetes. They augment the physiological activity of endogenous GLP-1 by promoting insulin secretion in a glucose-dependent manner, inhibiting glucagon release during hyperglycemia, slowing gastric emptying, and activating hypothalamic pathways to induce satiety. These effects lead to notable weight loss of around ~3 kg greater than placebo. Further, these agents improve glycemic control without causing major hypoglycemia^[13,14]^. Indeed, GLP-1 RAs are now FDA-approved for chronic weight management in obese or overweight patients^[15]^.

Interestingly, GLP-1 receptors are expressed in certain areas of the central nervous system responsible for drug- and food-related rewarding effects, such as the ventral tegmental area and the nucleus accumbens^[16]^ Consistent with this, GLP-1 RAs attenuate the drug reward for substances such as nicotine in animal models^[17,18]^.

Clinically, a recent trial consisting of prediabetic/overweight smokers given exenatide, a GLP-1 RA, in conjunction with nicotine patch improved abstinence rates along with lower drug cravings and withdrawals. Compared to only NRT, this combination also helped limit post-quit weight gain. After 6 weeks of therapy, the exenatide group had a 19.5% greater abstinence rate and weighed 5.6 pounds less than participants given a placebo^[19]^. Collectively, these data suggest that pharmacotherapies targeting the GLP1-R might attenuate nicotine-related reinforcement and help limit post-quit rise in body weight.

This narrative review aims to synthesize the existing evidence on the potential of GLP-1 RAs as a dual-action therapy for smoking cessation, with a particular focus on their ability to mitigate weight gain and improve overall outcomes.

### Methodology

A structured narrative literature search was conducted to identify studies examining the role of glucagon-like peptide-1 receptor agonists (GLP-1 RAs) in smoking cessation and weight management. Searches were performed in PubMed, ScienceDirect, and Google Scholar for publications between 1 January 2000 and 31 March 2025, using Boolean operators to combine terms such as (“GLP-1 receptor agonist” OR “GLP-1 RA” OR semaglutide OR liraglutide OR dulaglutide OR exenatide OR tirzepatide) AND (“smoking cessation” OR “nicotine dependence” OR “nicotine addiction” OR “tobacco use disorder”), as well as (“GLP-1 receptor agonist” OR “GLP-1 RA”) AND (“weight gain” OR obesity OR “weight management” OR “post-cessation weight gain”). Reference lists of key reviews and trials were also screened to capture additional studies. Eligible articles included preclinical animal studies, randomized controlled trials, observational studies, systematic and narrative reviews that addressed GLP-1 RAs in the context of nicotine dependence, smoking cessation, weight regulation, or obesity. Excluded were non-English publications, case reports, conference abstracts, grey literature, studies limited to diabetes treatment without relevance to smoking or weight outcomes, and duplicate reports without new findings. As this is a narrative rather than a systematic review, the potential for selection bias exists, and restriction to English-language studies may have introduced language bias; in addition, publication bias and author bias in interpretation are acknowledged. In accordance with the TITAN 2025 Guidelines for Ethical and Transparent Reporting of AI-Assisted Contributions in Academic Publishing, artificial intelligence (OpenAI ChatGPT, GPT-5, version 2025-09) was employed only for non-intellectual tasks, including language refinement, formatting assistance, and summarization of lengthy descriptions, while all intellectual tasks such as literature selection, interpretation, and synthesis of conclusions were performed by the authors, who take full responsibility for the scientific content^[20]^.

## The problem: smoking cessation and weight gain

The mechanisms underlying the association between smoking and reduced body weight are complex and not fully understood. Nicotine appears to play a key role in mediating these effects; however, cigarette smoking may also function as a behavioral alternative to eating, thereby decreasing overall food intake^[21]^.

Nicotine affects metabolism in several ways. First, it raises energy expenditure. It has been reported that there is a 6% increase in resting metabolic rate after 20 minutes of smoking^[22]^. It also causes increased energy expenditure during specific activities such as exercise^[23]^ Furthermore, one study found that smoking a cigarette increased daily energy expenditure by 10% (~140–200 kcal)^[24]^. This effect is two-fold as nicotine subsequently raises metabolic rate while also suppressing the compensatory increase in food intake that would typically follow. A 10% increase in metabolic rate, which corresponds to an additional 200 kilocalories expended per day, may seem insignificant. However, if caloric intake remains constant, this increase in energy expenditure could result in a body weight reduction of up to 10 kilograms over 1 year^[21]^.

Simultaneously, nicotine acts on the brain’s appetite centers to suppress hunger via binding to nicotinic acetylcholine receptors (nAChRs) in the hypothalamus. In particular, it activates pro-opiomelanocortin (POMC) neurons which trigger α-melanocyte-stimulating hormone release and melanocortin-4 receptor signaling to decrease food consumption^[25,26]^. Thus, quitting removes nicotine’s appetite-suppressing signals, leading to increased hunger, especially for calorie-rich foods^[27]^.

Smoking cessation also influences the regulation of appetite-related peptides, including hypothalamic neuropeptides such as neuropeptide Y and orexins, adipokines like leptin and adiponectin, and metabolic hormones including ghrelin and GLP-1. It results in increased food intake, decreased energy expenditure, and diminished satiety^[26,28,29]^.

Smoking involves a strong “hand-to-mouth” ritual, and people often replace that oral fixation with snacking^[3]^. Another contributing factor is compensatory eating wherein many quitters “replace” cigarettes with food or snacks. A study found that nicotine-withdrawn smokers tend to consume a higher overall caloric intake and exhibit a preference for more energy-dense foods compared to nonsmokers. This might be a coping mechanism or “self-medication” to deal with withdrawal-associated distress^[30,31]^. It has also been proposed that the abrupt decline in blood glucose levels during the initial days after quitting may contribute to withdrawal symptoms such as headaches, dizziness, and increased cravings for sugary foods, potentially leading to overeating to cope^[28,32,33]^. Thus, cessation can trigger overeating of high-calorie foods, driving rapid weight gain.

Excess weight is an established risk factor for a range of chronic health conditions, including CVD, type 2 diabetes, some malignancies, COPD, and mortality^[34–39]^. In a study comprising around 10 million participants across 239 studies, all-cause mortality was low for individuals with BMI 20-25. Notably, for each 5-unit increase in BMI above 25, the risk of mortality rose by 49% for cardiovascular disease, 38% for respiratory disease, and 19% for cancer^[36]^. Another study based on data from 12 European cohort studies found that each 5-unit rise in BMI was linked to a 34% increase in CVD mortality among men and a 29% increase among women^[40]^.

Literature concerning health consequences associated with post-quit weight or BMI gain are limited. A study involving data from three major U.S. cohorts found that the likelihood of developing type 2 diabetes increased in the subsequent 5–7 years following smoking cessation, especially among individuals who experienced more significant weight gain. However, this risk diminished over time. The diabetes incidence in former smokers aligned with that of never-smokers after approximately 30 years. These findings underscore that while short-term metabolic risks exist, the long-term benefits of quitting far outweigh them^[41]^.

Similarly, it has been suggested that additional weight could blunt some benefits of quitting on heart disease. Nevertheless, existing evidence indicates that smoking cessation significantly reduces the incidence of cardiovascular events, regardless of weight gain after quitting. A large cohort found former smokers had lower risk of myocardial infarction and stroke than continuing smokers, irrespective of post-cessation weight changes^[42]^.

Moreover, findings from a large prospective cohort study revealed that individuals who quit smoking experienced a significant reduction in all-cause mortality, even in the presence of weight or BMI gain. The study concluded that former smokers had a markedly lower risk of mortality compared to those who continued smoking, regardless of the extent of weight or BMI increase. Notably, post-cessation weight or BMI gain was not associated with elevated risks of CVD, COPD, type 2 diabetes, or cancer^[43]^.

Weight gain often yields social and psychological consequences. An overweight/obese individual may be stereotyped and stigmatized leading them to experience prejudice and discrimination from others. This can negatively affect their self-esteem^[44,45]^. This concern can adversely impact the success rate of quitting. In a study, about 34% of smokers indicated that they would cease treatment and return to smoking following weight gain^[46]^. This highlights that many quitters become discouraged by the additional body weight, which can undermine their satisfaction with quitting. In another study, post-quit weight gain increased the probability of relapsing at 6 months^[47]^. This suggests that weight gain can even trigger relapse, as the smoker may decide the trade-off (smoking vs obesity) is excessively high.

Ultimately, smokers may feel like they face a “trading one significant health risk for another challenge” dilemma. However, the data clearly favor quitting. Even though weight gain raises metabolic risks, the overall mortality and morbidity benefit of quitting is far larger. The disease risk from moderate weight gain is typically much lower than the damage done by continued tobacco and nicotine usage.

Given these challenges, the ideal therapeutic approach would concurrently alleviate nicotine withdrawal symptoms and mitigates post-cessation weight gain. Emerging evidence suggests that GLP-1RAs reduce voluntary nicotine consumption and seeking behaviors, while also preventing withdrawal-associated hyperphagia and subsequent weight gain. Additionally, recent findings indicate that these agents may ameliorate cognitive impairments and reduce depressive and anxiety-like symptoms, both of which are known to contribute to relapse during smoking cessation^[1]^.

Thus, GLP-1 RAs may offer exactly this dual benefit: by reducing appetite and also decreasing nicotine seeking behavior, they could help smokers quit without the usual weight penalty.

## GLP-1 RAs: a potential solution?

### Mechanism of action of GLP-1 RAs

#### Appetite regulation

Endogenous GLP-1, a gut hormone released in response to food intake, is mimicked by GLP-1 RAs. The hypothalamus and other brain areas that control appetite and energy balance have GLP-1 receptors, which these substances bind to a research claims that by affecting POMC neurons and inhibiting neuropeptide Y/agouti-related peptide (NPY/AgRP) pathways, activation of these receptors improves satiety signaling and decreases hunger^[48]^. The overall effect is a decreased desire to eat in between meals and a greater feeling of fullness after meals. Reduced food cravings, smaller portion sizes, and increased control over eating habits are common complaints from patients taking GLP-1 RAs. Because of these benefits, GLP-1 RAs are especially desirable for people who are susceptible to compensatory overeating when quitting smoking^[49]^.

#### Reward pathways

GLP-1 RAs may have an impact on the brain’s reward system in addition to controlling appetite. According to preclinical studies, these substances may alter dopaminergic transmission in the mesolimbic system, a region linked to addiction and reward processing. GLP-1 RAs have been shown by Herman & Schmidt^[1]^ to decrease nicotine-induced dopamine release in the nucleus accumbens, which attenuates the reinforcing effects of nicotine and may lessen cravings and the chance of relapse^[1]^. The addictive aspects of smoking can be addressed through new therapeutic approaches made possible by this neuromodulatory effect. GLP-1 RAs may be useful supplements to conventional smoking cessation treatments if they can reduce the brain’s reward response to nicotine^[50]^.

#### Metabolic effects

GLP-1 RAs have important metabolic effects in addition to their effects on the central nervous system. They help regulate blood glucose by enhancing the pancreatic β-cells’ glucose-dependent insulin secretion^[51]^. Additionally, GLP-1 RAs slow stomach emptying, which increases postprandial satiety and lowers caloric intake. According to some research, these substances may also boost energy expenditure and encourage fat oxidation, which could aid in weight loss and better metabolic health^[52]^.

**Figure 1.**
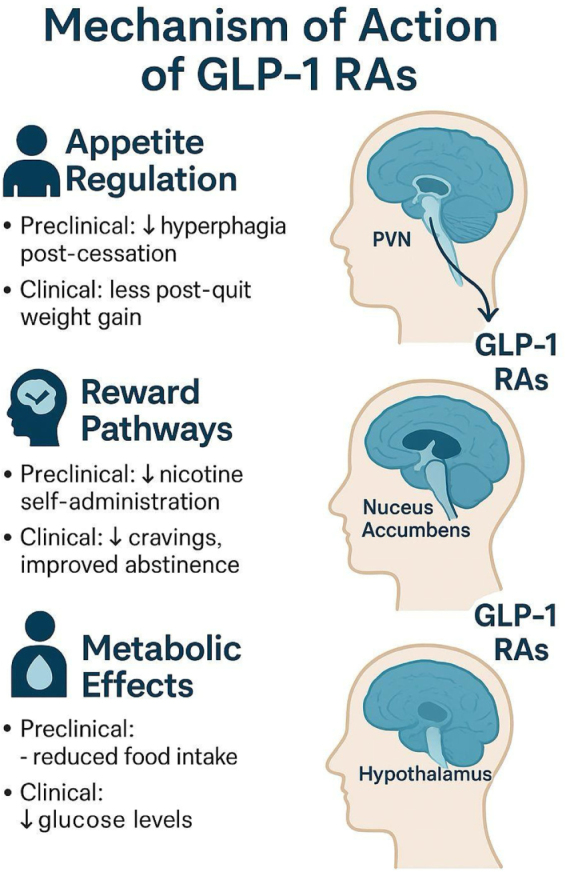
Mechanism of action of GLP-1 RAs: GLP-1 RAs reduce appetite by acting on hypothalamic pathways, influence reward processing via the mesolimbic system, and improve metabolic control by enhancing insulin secretion.

Figure 1 summarizes the overall mechanism of action of GLP-1 RAs.

### Preclinical and clinical evidence for GLP-1 RAs in smoking cessation

#### Animal studies

The use of GLP-1 RAs to help people quit smoking has received some initial support from animal models. GLP-1 analogs, like exendin-4, have been demonstrated in rodent studies to decrease nicotine self-administration and to lessen withdrawal symptoms like agitation and anxiety. According to these results, GLP-1 RAs may help with the psychological and physiological aspects of nicotine dependence^[53]^.

#### Human trials

Although evidence from human studies is still limited, two important trials have begun to explore the role of GLP-1 RAs in smoking cessation. The first is the SKIP trial protocol published by Lengsfeld *et al*, which outlined a single-center, randomized, placebo-controlled, double-blind, parallel-group design to test dulaglutide as an adjunct to behavioral therapy^[50]^. The protocol specified once-weekly subcutaneous injections of dulaglutide 1.5 mg for 12 weeks, in combination with standard behavioral counseling, with the aim of improving abstinence rates and limiting post-cessation weight gain. This trial provided the first structured framework for evaluating GLP-1 RAs in this context^[50]^.

Building on this, Lüthi *et al* reported results from the first completed randomized, double-blind, placebo-controlled trial in 255 adult smokers attempting to quit^[54]^. Participants were randomized to receive dulaglutide 1.5 mg once weekly via subcutaneous injection or placebo, in addition to behavioral counseling, for up to 12 months. Although the primary endpoint 12-month smoking abstinence did not reach statistical significance, secondary outcomes were encouraging: dulaglutide was associated with reduced nicotine cravings, less post-cessation weight gain, and higher self-reported satisfaction with the quitting process^[54]^.

Adverse effects were consistent with the known safety profile of GLP-1 RAs. The most common were gastrointestinal symptoms, including nausea, vomiting, diarrhea, and abdominal discomfort, which were most pronounced in the first 1–3 weeks. About 90% of participants in the dulaglutide arm reported some GI effects, compared with 81% in placebo, and some required supportive therapy such as antiemetics or proton pump inhibitors. Other frequently reported adverse events included mild upper respiratory tract infections. A small number of participants withdrew due to adverse effects (10 in the dulaglutide group vs six in placebo), but importantly, no treatment-related serious adverse events or unexpected safety signals were observed^[50,54]^.

Despite these promising early findings, both studies have limitations, including the single-center design, modest sample size, short follow-up in SKIP, reliance on self-reported outcomes, and lack of multicenter validation. Larger, well-designed randomized controlled trials with longer follow-up are essential to confirm efficacy, better define the long-term safety profile, and establish cost-effectiveness before GLP-1 RAs can be recommended as part of routine smoking cessation programs^[50,54]^.

#### Mixed results and need for further research

The picture painted by the current corpus of research is conflicting but positive. More thorough research is needed, even though animal data are reliable and preliminary human trials point to possible advantages. Research in this area is difficult due to the complexity of smoking behavior, interindividual variability in treatment response, and the dual objectives of weight control and cessation. GLP-1 RAs should be used in conjunction with proven smoking cessation techniques rather than in place of them until more conclusive evidence is available.

### Evidence for the weight management effects of GLP-1 RAs

#### Clinical trials

GLP-1 RAs have a well-established effect on weight loss when used to treat type 2 diabetes and obesity. Patients treated with GLP-1 RAs consistently show significant weight reductions, according to clinical trials, including those compiled by Quinter *et al*^[54]^. Depending on the agent and length of treatment, patients who are obese or overweight typically lose 5%–15% of their starting body weight. For instance, some studies have linked semaglutide 2.4 mg weekly to weight loss of over 15%, which is similar to the amount attained with bariatric surgery^[55]^. Table 1 summarizes the preclinical and clinical studies on GLP-1 RAs in smoking cessation.

**Table 1:** Summary of preclinical and clinical studies on glucagon-like peptide-1 receptor agonists (GLP-1 RA’s) in smoking cessation

Study type	Model/population	GLP-1 RA tested	Key findings	Reference
Preclinical (rodent)	Mice	Exendin-4	Reduced nicotine-induced locomotor activity, accumbal dopamine release, and conditioned place of preference.	Egecioglu *et al*. (2013)
Preclinical (rodent)	Rats	Exendin-4	Reduced reinstatement of nicotine- and heroin-seeking behavior; attenuated withdrawal symptoms.	Tuesta *et al* (2017); Douton *et al* (2020)
Clinical (pilot RCT)	255 adult smokers	Liraglutide	Reduced nicotine cravings, less weight gain, higher satisfaction with cessation, but no significant effect on abstinence rate.	Herman & Schmidt (2024)
Clinical (pilot RCT)	Overweight/prediabetic smokers using nicotine patch	Exenatide	Improved abstinence rates (+19.5% vs placebo) reduced cravings/withdrawals, less post-cessation weight gain (−5.6 lbs).	Yammine *et al* (2021)
Clinical (RCT, 12 months)	Smokers in cessation program	Dulaglutide	Higher abstinence rates vs placebo sustained effect at 12 months.	Lüthi *et al* (2024)
Ongoing trial	Smokers (planned)	GLP-1 analogues (SKIP trial)	Protocol designed to evaluate GLP-1 RA’s as a cessation aid.	Lengsfeld *et al* (2023)

#### Comparison of different GLP-1 RAs

Among GLP-1 RAs, semaglutide, liraglutide, and tirzepatide are FDA-approved for chronic weight management. Other agents such as exenatide have demonstrated efficacy but are not specifically FDA-approved for weight loss indications. Although semaglutide has the strongest effects on weight loss, it may also have a higher risk of gastrointestinal adverse effects like nausea and vomiting. When taken daily, ligarglutide has a more gradual onset of effects but a slightly lower efficacy. Because of its shorter half-life and less desirable side effect profile, exenatide is not as frequently used for weight loss. Patient preference, comorbid conditions, cost, and tolerance for side effects are frequently factors in selecting the best agent^[56]^.

#### Long-term weight maintenance

Maintaining weight loss is a significant obstacle in the treatment of obesity. GLP-1 RAs have been shown to support weight loss, particularly when used consistently over an extended period of time^[14]^. According to data from extension studies, patients who stick with their treatment have a lower chance of gaining back the weight they lost, which may indicate a long-lasting effect on metabolic rate and appetite control^[57]^. This long-term benefit is especially important for people who are quitting smoking because it gives them a way to control their weight in the months and years after quitting, when there is a high chance of relapsing^[54]^.

## Benefits of GLP-1 RAs in addressing post-cessation weight gain

One of the best ways to improve long-term health outcomes is to stop smoking, but many people are reluctant to do so because they worry about gaining weight later. Usually brought on by metabolic alterations and an increase in appetite after quitting nicotine, this weight gain can discourage attempts to quit and is a major factor in relapse for many people^[58]^. Initially created for glycemic control in type 2 diabetes, GLP-1 RAs have become a promising pharmacological treatment that could allay this worry. In addition to helping people lose weight, GLP-1 RAs have a number of special benefits that make them an effective way to improve smoking cessation results^[13]^.

### Targeting both nicotine addiction and weight gain simultaneously

Targeting both nicotine addiction and weight gain, GLP-1 RAs’ dual action on the addictive and metabolic aspects of quitting smoking is one of their biggest advantages over other weight-management techniques^[1]^. Conventional smoking cessation treatments like varenicline, bupropion, or NRT mostly address withdrawal symptoms and decrease nicotine cravings, but they don’t do much to address the weight gain that comes with it. In contrast, GLP-1 RAs may also affect brain reward pathways in addition to suppressing appetite and reducing caloric intake^[59]^. According to new research, GLP-1 RAs may be able to alter dopaminergic activity in the mesolimbic system, which is a region implicated in the reinforcement and reward mechanisms linked to addictive behaviors. This increases the possibility that GLP-1 RAs will reduce nicotine cravings and mood disorders brought on by withdrawal, increasing the chance of quitting smoking successfully. GLP-1 RAs provide a holistic approach that may be more successful than methods that focus on just one area by concurrently addressing mechanisms linked to appetite and addiction^[60]^.

### Potential for improved adherence to smoking cessation programs

Concerns about weight gain are frequently mentioned as the cause of relapse following smoking cessation, particularly among women and those who already have weight management issues^[61]^. GLP-1 RAs’ capacity to reduce weight gain after cessation may allay these anxieties and increase motivation to stop. People may be more inclined to participate in cessation programs if they believe they can stop smoking without incurring the “cost” of putting on weight^[62]^. Furthermore, patients may feel more empowered and inspired to stick with long-term behavioral changes if they have early success quitting smoking and maintaining their weight. This can enhance adherence to more comprehensive lifestyle interventions, like more exercise and better eating habits, as well as to cessation therapies^[63]^. When paired with behavioral support and counseling, GLP-1 RAs may prove to be a compelling adjunct therapy in clinical settings for reinforcing positive behavioral outcomes^[64]^.

### Additional health benefits

Beyond glycemic control and weight loss, GLP-1 RAs are linked to a number of cardiometabolic advantages. Drugs like semaglutide and liraglutide have shown cardiovascular protective effects in people with type 2 diabetes, including a decrease in major adverse cardiovascular events^[65]^. These advantages are especially pertinent when it comes to quitting smoking because ex-smokers frequently continue to have a higher risk of cardiovascular disease years after they stop^[65]^. Patients may benefit from additional protection against cardiovascular disease during this crucial transitional phase by including GLP-1 RAs in their cessation plan. GLP-1 RAs are especially helpful for patients with prediabetes or metabolic syndrome because they also enhance insulin sensitivity, lower fasting glucose levels, and improve glycemic control^[66]^. Using GLP-1 RAs during the post-cessation phase may lower the risk of insulin resistance and type 2 diabetes, as smoking is also a risk factor for these conditions. This makes GLP-1 RAs a more comprehensive tool for enhancing long-term metabolic health as well as a weight-management aid^[67]^.

### Improving overall smoking cessation success rates

The ability of the individual to sustain abstinence over time is ultimately what determines whether smoking cessation efforts are successful. Self-esteem and psychological well-being can be significantly impacted by addressing weight gain, which is a common cause of relapses^[68]^. People may feel more in control of their health and happier with the results of quitting smoking if they stop smoking without gaining a lot of weight. This can boost motivation and raise the possibility of long-term abstinence^[69]^. Avoiding weight gain can lessen the internal conflict that frequently arises between wanting to stop smoking and fearing its physical consequences. Self-image is a crucial factor in health behavior change^[61]^. GLP-1 RAs can increase both short-term and long-term quit rates by addressing one of the main psychological obstacles to quitting. By assisting more people in quitting smoking and improving their cardiometabolic outcomes, their use may have wider public health benefits^[50]^.

### Challenges and considerations

Besides their promise, use of GLP-1 receptor agonists (GLP-1 RAs) is also associated with a number of significant challenges and issues. Nausea, vomiting, diarrhea, and constipation are among the most common side effects of these medications and are extraordinarily commonly observed adverse events^[70]^. They are typically dose dependent in nature and can oftentimes be avoided by stepwise dose titration and dietary modification^[71]^. However, severity and frequency may vary depending on different agents, though semaglutide has often been associated with higher rates of gastrointestinal adverse events compared to other GLP-1 RAs^[72]^.

Also to be mentioned are rare but serious side effects such as acute pancreatitis, gallbladder disease, and a possible risk of medullary thyroid carcinoma^[72]^. Though uncommon, they go to identify correct patient selection as well as surveillance with therapy^[73]^. GLP-1 RAs are also contraindicated in patients with personal or family history of medullary thyroid carcinoma or multiple endocrine neoplasia syndrome type 2, which requires clinical awareness^[73,74]^.

The second essential challenge is the expense and availability of GLP-1 RAs. The drugs are quite costly and possibly not affordable to most people with limited insurance coverage^[74]^. While there are some health insurance policies that cover the drugs, high out-of-pocket costs can deter their use on a large scale^[75]^. Economic modeling indicates that although GLP-1 RAs actually decrease obesity conditions, their application in wider populations would be a huge economic burden for health systems unless cost-saving strategies are utilized^[76]^. In addition, socioeconomic inequalities also further worsen access barriers, especially among racial and ethnic minorities who might already experience access issues in obesity and smoking cessation treatment^[77]^.

Lifestyle treatment in addition to pharmacotherapy is essential in order to obtain the best results. GLP-1 RAs are best utilized with behavioral therapy supporting healthy food and exercise habits^[78]^. Pharmacologic and lifestyle interventions combined have been found by clinical trials to offer higher weight loss and cardiometabolic results compared to drug monotherapy^[78]^. Specialized diet therapy typically includes high-protein, low-glycemic-index diets and organized exercise regimens, which are beneficial for weight management and also with adherence to treatment^[79]^.

While showing promising metabolic effect, evidence for the application of GLP-1 RAs in smoking cessation is limited. Direct evaluation of efficacy for this purpose is missing, and there is mainly preclinical and indirect human evidence^[17]^. Initial evidence indicates that GLP-1 RAs may have an impact on reward circuits to nicotine dependence but needs well-powered randomized controlled trials to determine this and assess clinical application^[17]^.

### Comparing other smoking cessation agents

Although GLP-1 RAs seem promising to aid smoking cessation, the evidence base for established pharmacotherapies is considerably stronger. Current pharmacotherapy options include varenicline, NRT, and bupropion.

Among these, varenicline remains the most effective. It has a dual mechanism. Its partial agonism stimulates low-level dopamine release whilst its antagonist activity blocks inhaled nicotine from binding to these receptors, thereby attenuating the pleasure derived from smoking^[80]^. Varenicline has consistently demonstrated the greatest efficacy among approved monotherapies. A meta-analysis reported a pooled risk ratio of 2.83 for continuous abstinence at 52 weeks compared with placebo^[81]^, while a network meta-analysis similarly found that varenicline nearly doubled the likelihood of sustained cessation^[82]^.

Bupropion acts as a norepinephrine–dopamine reuptake inhibitor. One study found that approximately one in five smokers successfully cease and remain non-smoking at 1 year with bupropion therapy^[83]^. While less effective as varenicline, bupropion significantly improved abstinence relative to control, corresponding to roughly three additional quitters per 100 treated individuals^[82]^. Importantly, bupropion offers a modest weight-reducing effect^[84]^ that may help offset post-cessation weight gain.

NRT delivers nicotine via non-combustible methods such as patches, gums, or lozenges to reduce withdrawal symptoms and cravings. Evidence indicates that all forms of NRT improve quit rates, increasing the likelihood of successful cessation by approximately 50%–70%^[85]^. In addition, a meta-analysis found that NRT also helps reduce cigarette consumption among individuals not ready to quit entirely^[86]^.

The EAGLES trial assessed the safety and efficacy of several smoking cessation therapies, including bupropion, varenicline, the nicotine patch, and placebo. Participants treated with varenicline achieved more than threefold higher odds of quitting compared with placebo. When compared with other active treatments, varenicline also demonstrated superior efficacy, yielding significantly greater quit rates than both the nicotine patch and bupropion. Nevertheless, both bupropion and the nicotine patch outperformed placebo, reinforcing their established effectiveness in smoking cessation, albeit with smaller effect sizes than varenicline^[87]^.

Compared to GLP-1 RAs, standard treatments are often more affordable and accessible. Agents like bupropion, varenicline and NRT are often covered by insurance, e.g. Medicare^[88]^. In contrast, GLP-1 RAs typically require prior authorization, often with strict criteria such as a BMI ≥30, documented failure of other weight-loss medications, or the presence of comorbidities like uncontrolled hypertension^[89]^. Furthermore, traditional agents have a well-understood safety profile. However, for GLP-1 RAs, the adverse effects linked specifically for smoking along with long-term abstinence data are not clearly characterized, warranting further research.

The overall profiles and easy access of established pharmacotherapies suggest that GLP-1 RAs may be best positioned as an adjunctive therapy rather than a replacement for current treatments. The synergist effect of combining a highly effective abstinence drug (like varenicline) with an intervention that mitigates the primary deterrent of weight gain (a GLP-1 RA) holds considerable promise for improving long-term success in quitting, particularly for patients with comorbid metabolic disorders.

## Limitations

This review has several limitations. First, the majority of available evidence regarding GLP-1 RAs in smoking cessation comes from preclinical studies and small-scale or pilot human trials, limiting the generalizability of findings to broader and more diverse populations. Second, heterogeneity exists in study populations, intervention type (e.g. type of GLP-1 RAs), dose, and treatment duration, which makes direct comparisons challenging and limits the ability to draw consistent conclusions. Third, most trials have short follow-up periods, and long-term efficacy, safety data, and relapse rates in the context of smoking cessation remain lacking. Fourth, many studies depend on self-reported smoking outcomes, which are subject to biases, further weakening the strength of the evidence. Fifth, potential publication bias may overrepresent positive findings. Finally, as this is a narrative review rather than a systematic review, the possibility of selection bias in included studies cannot be excluded.

Taken together, these limitations underscore the need for larger, multicenter randomized controlled trials with longer follow-up periods, standardized interventions, and biomarkers of smoking cessation such as Nicotine Metabolite Ratio^[90]^ to more definitively establish the role of GLP-1 RAs in this context.

## Future directions

The future should be the performance and design of big, randomized controlled trials to adequately assess the safety and efficacy of GLP-1 RAs for weight loss and cessation of smoking during the post-cessation phase. The trials must determine which subpopulations are most likely to benefit and the best dose and treatment duration.^[19]^ Also, there is a strong reason for investigating combination approaches, including the combination of GLP-1 RAs with cognitive behavior therapy or NRT, to manage both the metabolic and neuropsychological components of smoking dependence^[1]^. Further, long-term follow-up studies are necessary to assess the long-term sustainability of smoking cessation, weight reduction, and total health outcomes with GLP-1 RA therapy^[72]^.

These information will be crucial to evaluate cost-effectiveness as well as to establish clinical guideline. Personalized medicine strategies provide another promising direction, in which therapy may be individualized according to genetic background, smoking habit, metabolic status, and behavioral characteristics^[91]^. Stratification of such kind can maximize effectiveness, reduce side effects, and enhance long-term smoking cessation program compliance with the inclusion of GLP-1 RA therapy^[91]^.

## Conclusion

Smoking cessation remains a significant challenge, not only because of nicotine dependence but also due to the weight gain that often follows quitting. This post-cessation weight gain is a well-recognized barrier that can reduce motivation, lower self-esteem, and even trigger relapse. Metabolic and behavioral changes after nicotine withdrawal frequently lead to increased food intake and reduced energy expenditure, replacing one health risk tobacco use, with another obesity. GLP-1 RAs, originally developed for type 2 diabetes and later approved for obesity management, have recently been explored as a potential adjunct in smoking cessation. These agents regulate appetite and support weight loss, while also influencing neural reward pathways involved in addiction. Preclinical studies and a small number of early clinical trials suggest potential benefits in reducing nicotine cravings and limiting post-cessation weight gain. However, the current evidence is preliminary, based on small sample sizes, short follow-up, and reliance on self-reported outcomes, and should therefore be interpreted with caution. While GLP-1 RAs are promising, their role in smoking cessation remains investigational. Future large-scale, long-term randomized controlled trials are needed to confirm efficacy, assess safety, and evaluate cost-effectiveness compared with established cessation agents. Until then, GLP-1 RAs should be regarded as a potential but unproven strategy.

## Peer and provenance statement

Externally peer reviewed; not commissioned.
